# Effects of Molecular Crowding on the Dynamics of Intrinsically Disordered Proteins

**DOI:** 10.1371/journal.pone.0049876

**Published:** 2012-11-26

**Authors:** Elio A. Cino, Mikko Karttunen, Wing-Yiu Choy

**Affiliations:** 1 Department of Biochemistry, The University of Western Ontario, London, Ontario, Canada; 2 Department of Chemistry, University of Waterloo, Waterloo, Ontario, Canada; Weizmann Institute of Science, Israel

## Abstract

Inside cells, the concentration of macromolecules can reach up to 400 g/L. In such crowded environments, proteins are expected to behave differently than *in vitro*. It has been shown that the stability and the folding rate of a globular protein can be altered by the excluded volume effect produced by a high density of macromolecules. However, macromolecular crowding effects on intrinsically disordered proteins (IDPs) are less explored. These proteins can be extremely dynamic and potentially sample a wide ensemble of conformations under non-denaturing conditions. The dynamic properties of IDPs are intimately related to the timescale of conformational exchange within the ensemble, which govern target recognition and how these proteins function. In this work, we investigated the macromolecular crowding effects on the dynamics of several IDPs by measuring the NMR spin relaxation parameters of three disordered proteins (ProTα, TC1, and α-synuclein) with different extents of residual structures. To aid the interpretation of experimental results, we also performed an MD simulation of ProTα. Based on the MD analysis, a simple model to correlate the observed changes in relaxation rates to the alteration in protein motions under crowding conditions was proposed. Our results show that 1) IDPs remain at least partially disordered despite the presence of high concentration of other macromolecules, 2) the crowded environment has differential effects on the conformational propensity of distinct regions of an IDP, which may lead to selective stabilization of certain target-binding motifs, and 3) the segmental motions of IDPs on the nanosecond timescale are retained under crowded conditions. These findings strongly suggest that IDPs function as dynamic structural ensembles in cellular environments.

## Introduction

Macromolecular crowding and confinement can have significant impacts on the behaviors of proteins in cellular environments. Inside of cells, the concentration of macromolecules can reach up to 400 g/L [1], [2]. The cumulative excluded volume from all macromolecules inside of cells is commonly referred to as macromolecular crowding [3], [4]. The large volume occupied by macromolecules in the cellular environment exerts nonspecific forces on surrounding molecules [3]. It is well documented that these forces can have significant effects on the behaviors of proteins [5]–[7].

Experimental studies have demonstrated that molecular crowding can affect protein structure and function. For example, at low pH, cytochrome c adopts an unfolded form. When the crowding agent dextran is added to the sample, the protein transitions into a near-native molten globule state [8]. Crowding has also been shown to enhance the activity of phosphoglycerate kinase (PGK) in vitro. At a mild concentration of Ficoll 70 (100 g/L), the enzymatic activity of PGK was found to increase by more than 10 fold (after the viscosity effect was taken into account), possibility due to the large-scale of conformational changes induced by the crowders [9]. In another study, Stagg et al. [10] investigated effects of crowding on the structure and stability of both the native and denatured states of Flavodoxin. Interestingly, their experimental and computer simulation results indicate that the presence of a high concentration of Ficoll 70 in solution increased the thermal stability and secondary structure content of the native-state ensemble, but had relatively minor effects on the denatured state [10].

The crowded environment in cells also alters the diffusional behavior of proteins, and thus their rates of folding, association with other molecules and intracellular transport [11], [12]. A recent work by Leduc et al. [13] suggested that different motor proteins, such as kinesins, process distinct molecular properties in order to operate effectively in the crowded cellular environments. Macromolecular crowding has also been proposed to be one of the possible factors that regulate the phosphorylation of ERK kinase in cells. Aoki et al. [14] demonstrated that under crowded conditions, the phosphorylation of ERK could switch from the distributive to processive mode. Further, experimental and molecular simulation studies suggested that crowding plays a key role in human diseases that are related to protein aggregation and fibril formation [15]–[17]. For instance, the amyloid formation of human and bovine prion proteins are significantly enhanced even at mild concentration (150–200 g/L) of Ficoll 70. Intriguingly, the amyloid formation of rabbit prion protein is inhibited by crowding agents [17], [18].

The effects of macromolecular crowding on the structure and dynamics of IDPs, on the other hand, are less explored. These proteins lack stable tertiary structures and can be very flexible under non-denaturing conditions. The functions of IDPs are intimately related to their dynamics [19]. It has been proposed that proteins with disordered regions have larger capture radius for targets, therefore, enhancing the binding rates by the so-called “fly-casting” mechanism [20]. Flexibility of IDPs also governs the affinity of target recognition. The high entropic cost of disorder-to-order transition upon binding needs to be compensated by specific interactions formed in the interface with target. Therefore, IDPs frequently associate with binding partners through low affinity but highly specific interactions, which are important for their functions in signal transduction and cell cycle control [21], [22]. Another important link between protein flexibility and function is the rate of inter-conversion between conformers. An IDP exists as an ensemble of conformers in equilibrium [23]–[25]. Different structures in the ensemble can participate in the interactions with distinct targets; therefore, the rate of exchange between conformers can have significant impact on the protein function [26], [27]. Further, recent studies show that some IDPs employ multiple linear motifs to engage in a dynamic equilibrium with a target, resulting in ultra-sensitivity of binding [28]–[30]. Undoubtedly, protein flexibility plays a critical role in this polyvalent mode of binding [29].

There are several studies of macromolecular crowding effects on the structure of IDPs. The results, however, are not conclusive. For instance, FlgM is disordered in dilute buffer solutions, but gains structure in its C-terminal half when studied in cells or in solutions with high concentration of glucose [31]. On the other hand, Flaugh and Lumb reported that neither the disordered C-terminal activation domain of c-Fos nor the kinase-inhibition domain of p27^Kip1^ undergo any significantly conformational change in the presence of dextran or Ficoll [32]. By using small-angle neutron scattering techniques, Johansen et al. [33] demonstrated that the disordered N protein of bacteriophage λ adopts more compact conformations even in the presence of relatively low concentration of crowding agents (∼65 g/L of BPTI protein). A recent work by Tompa and co-worker [34], however, shows that molecular crowding caused only minor structural changes to three IDPs (α-casein, MAP2c and p21^Cip1^). The authors suggested that retaining dynamics under crowded conditions is a functional requirement of IDPs.

Further experimental studies of the macromolecular crowding effects on IDPs are important for increasing our understanding of how these proteins behave in cellular environments. These studies will also facilitate the development of computational models that can be used to explain and predict the behaviors these proteins under crowded conditions [5], [34], [35]. We focus on assessing the effects of macromolecular crowding on the dynamics of IDPs in residue-specific manner using NMR spin relaxation experiments. Three IDPs with different extents of residual structure under dilute buffer conditions were studied. Further, by using one of the IDPs (ProTα) as a representative case, based on an MD simulation, we proposed a model to correlate the observed changes in relaxation rates to the possible alteration in protein motions under crowding conditions. ProTα is a ubiquitously expressed, highly acidic IDP that is involved in multiple biological functions [36]–[38]. Our recent studies demonstrated that ProTα is largely disordered with minimal residual structure present under non-denaturing conditions [39], [40]. Although ProTα adopts an extended structure, it can convert to more compact conformations in the presence of zinc ions [40]. Another IDP used in this study is Thyroid Cancer 1 (TC-1), which was first found to be overexpressed in thyroid cancer [41], [42]. TC-1 is a basic protein and is a positive regulator of the Wnt/β-catenin signaling pathway [42]–[44]. It competes with β-catenin on binding to Chibby (Cby) and therefore inhibits the antagonistic action of Cby on β-catenin mediated transcription [44], [45]. Even though TC-1 is classified as an IDP, structural characterization shows that while the N-terminal half of the protein is largely unstructured, high helical propensity is present in the C-terminal part [42], [46]. α-synuclein, a well-studied IDP that has been found to be the main structural component of Lewy body fibrils found in patients with Parkinson’s disease [47], was also included in this study to add additional depth to our approach. α-synuclein is natively disordered in its soluble form, but is able to self-associate to form insoluble aggregates that have considerable structure [47]. In-cell NMR experiments have shown that the periplasmic environment in *Escherichia coli* prevents α-synuclein from undergoing a conformational change that is detected in dilute buffer conditions, indicating that the crowding acts to keep α-synuclein disordered [48]. In addition to the IDPs mentioned above, we also assessed the crowding effect on a well-studied globular protein, Ubiquitin, for comparison. By performing NMR relaxation measurements on these proteins we aim to determine how the dynamics of IDPs with different structural characteristics can be affected by macromolecular crowding.

## Materials and Methods

### Protein Expression and Purification

Uniformly ^15^N labeled ProTα (human isoform 2), TC-1 (human) and α-synuclein (human isoform 1) were expressed in *Escherichia coli* BL21 (DE3) cells grown in minimal M9 medium containing ^15^NH_4_Cl (Cambridge Isotope Laboratories) as the sole nitrogen source. ^15^N/^13^C labeled TC-1 was expressed as above except with ^13^C_6_-D-glucose (Isotec) as the sole carbon source. ProTα was purified using the method described by Yi et al. [39]. The N-terminally His tagged TC-1 protein was extracted from inclusion bodies using 6 M guanidine hydrochloride and purified by affinity chromatography using Ni Sepharose™ 6 Fast Flow beads (Amersham Biosciences) [46]. The plasmid carrying the α-synuclein cDNA was kindly supplied by Dr. Pielak at the University of North Carolina-Chapel Hill. The protein was purified by osmotic shock, using a procedure similar to the one reported by Shevchik et al. [49], followed by boiling and cooling steps similar to [39]. The protein was then precipitated out of solution with 60% saturated solution of ammonium sulfate. Lyophilized ^15^N labeled human Ubiquitin was kindly supplied by Dr. Gary Shaw’s lab at the University of Western Ontario.

### NMR Spectroscopy

All NMR experiments were performed at 25°C on a Varian Inova 600 MHz spectrometer (UWO Biomolecular NMR Facility) with an xyz-gradient triple resonance probe. The experiments were performed in the presence and absence of 160 g/L, and several used 400 g/L, Ficoll 70 (Sigma) or Dextran 70 (Sigma). Each NMR sample contained 10% D_2_O and trace sodium 2,2-dimethyl-2-silapentane-5-sulfonate (DSS, Sigma) for chemical shift referencing. Data was processed with NMRPipe [50] and spectra were visualized with NMRViewJ [51].

^1^H-^15^N HSQC spectra were collected using 0.2 mM ^15^N-labeled ProTα, TC-1 and α-synuclein samples and 1 mM Ubiquitin samples in the presence or absence of crowding agent. Backbone amide resonance assignments of ProTα, TC-1, α-synuclein and Ubiquitin were obtained from [40], [46], [52], [53]. The triple-resonance CBCA(CO)NH experiment was carried out using 0.3 mM TC-1 samples in the presence and absence of 160 g/L Ficoll 70 (Sigma) for ^13^C chemical shift assignments.

Backbone ^15^N longitudinal relaxation rate (*R_1_*), relaxation rate in rotating frame (*R_1ρ_*), and steady-state ^1^H-^15^N NOE experiments were performed using 0.2 mM of ^15^N-labeled ProTα, and TC-1 samples and 1 mM Ubiquitin sample in the presence and absence of crowding agent in their corresponding buffers. *R_1_* experiments were performed with delay times 10–640 ms for ProTα and TC-1 and 10–500 ms for Ubiquitin. *R_1ρ_* experiments employed delay times between 10 and 150 ms for all proteins. The relax program [54], [55] was used for two-parameter exponential curve fitting of peak intensities from the *R_1_* and *R_1ρ_* data, and the calculation of *R_1_* and *R_1ρ_* relaxation rates and their associated errors. ^15^N transverse relaxation rate (*R_2_*) values were calculated using the *R_1_* and *R_1ρ_* rates and the offset between the resonance and carrier frequency (Δω) in hertz, using the equation.
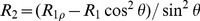
(1)where tan*θ* = B_SL_/Δω. B_SL_ ( = 1.5 kHz) was the spin-lock field used in the *R_1ρ_* experiments. ^1^H-^15^N steady-state NOEs were obtained from the ratio of peak intensities of spectra recorded with and without proton saturation. Seven and 12 s delays between scans were used for the saturated and non-saturated spectra respectively and 5 s saturation periods were used. Errors were estimated based on the ratios of background noise to the signals in the spectra.

### MD Simulations

We conducted an atomistic MD simulation of ProTα in its free state in order to help to interpret the NMR relaxation measurements. The starting structure was generated based upon the amino acid sequence of ProTα (human isoform 2) by simulated annealing using the Crystallography & NMR System (CNS) software package [56].

The simulation was performed using GROMACS (GROningen MAchine for Chemical Simulations) version 4 [57] with the GROMOS96 53a6 united atom force-field parameter set [58], [59]. This force field has been shown to perform well in simulations of disordered proteins and membrane proteins [60]–[62]. Protonation states of ionizable residues were assigned to their most probable state at pH 7. The starting structure was centered in a cubic box with a side length of 20 nm and periodic boundary conditions were applied. The system was solvated with simple point charge (SPC) water [63]. Sodium (Na^+^) and chloride (_Cl_^-^) ions were added to make the system charge neutral and bring the salt concentration to 0.1 M. The system contained 265474 water molecules, 525 sodium and 482 chloride ions. MD simulations were performed at constant number of particles, pressure and temperature (NPT ensemble). Protein and non-protein atoms were coupled to their own temperature baths, which were kept constant at 310 K using the Parrinello-Donadio-Bussi algorithm [64]. Pressure was maintained isotropically at 1 bar using the Parrinello-Rahman barostat [65]. The time constants for temperature and pressure coupling were 0.1 and 0.5 ps, respectively. Prior to the production run, the energy of the system was minimized using the steepest descents method, followed by 2 ps of position-restrained dynamics with all non-hydrogen atoms restrained with a 1000 kJ mol^−1^ force constant. The timestep was set to 2 fs. Initial atom velocities were taken from a Maxwellian distribution at 310 K. All bond lengths were constrained using the LINCS algorithm [66]. Cut-off of 1.0 nm was used for Lennard-Jones interactions and the real part of the long-range electrostatic interactions, which were calculated using the Particle-Mesh Ewald (PME) method [67]. For a recent review on the different methods and the importance electrostatics in simulations of biological systems, see [68]. Dispersion corrections were applied for energy and pressure. 0.12 nm grid-spacing was used for PME. The MD simulation was run for 427 ns and the last 400 ns were used for analysis. During this time, temperature, pressure and potential energy values remained stable and fluctuated around their averages, without systematic drift, indicating that the system was well equilibrated.

### MD Simulation Analysis

Autocorrelation functions of backbone ^1^H-^15^N bond vectors of ProTα were extracted from the MD trajectory (region 27–427 ns) (without the removal of overall tumbling) using the g_rotacf tool in GROMACS [57]. Each autocorrelation function was fitted to two-, three-, or four-exponential decay curves [69]–[71] as shown in equation (2):
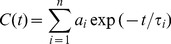
(2)where *C(t)* is the autocorrelation function at time *t*, n = 2, 3, or 4, *a_i_* and *τ_i_* are the amplitude and time constant of the *i* ^th^ exponential decay term. The fitted autocorrelation functions were then used to calculate the spectral density *J(ω)* by analytical Fourier transformation [69]–[71]:



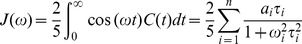
(3)To evaluate whether the multi-exponential model *j* with more parameters statistically outperforms model *i* in fitting the autocorrelation functions, the F-ratio of statistical F-test were calculated using the following equation:
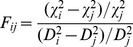
(4)where 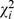
(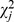) and *D_i_* (*D_j_*) are the sum of square deviations and degrees of freedom of model *i* (model *j*), respectively.

## Results

### IDPs Remain Disordered Under Crowded Environments

**Figure 1:**
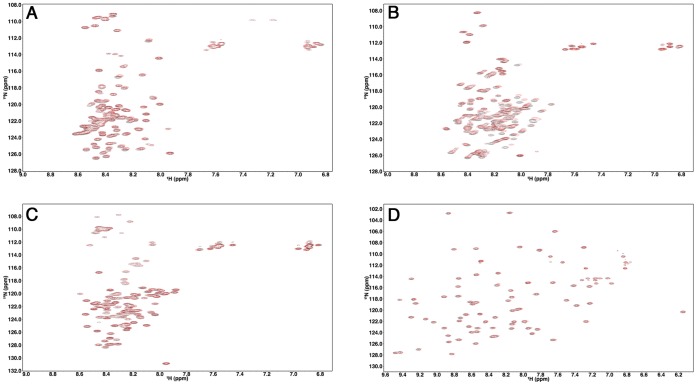
^1^H-^15^N HSQC spectra of ProTα, TC-1, α-synuclein and Ubiquitin in the absence and presence of 160 g/L Ficoll 70. ProTα (A), TC-1 (B), α-synuclein (C) and Ubiquitin (D) spectra were collected in 40 mM HEPES pH 6.8, 10 mM sodium acetate pH 5, 50 mM sodium phosphate pH 7 and 10 mM sodium acetate pH 5 respectively in the absence (black) and presence of 160 g/L Ficoll 70 (red).

To study the effect of macromolecular crowding on the structure and dynamics of IDPs, Ficoll 70, a commonly used crowding agent, was added to the protein samples to mimic the cellular environment [6]. First, ^1^H-^15^N HSQC spectra of ProTα, TC-1, α-synuclein, and Ubiquitin, acquired in the absence and presence of 160 g/L of Ficoll 70, were compared. Intriguingly, the spectra of the three IDPs all display narrow peak dispersions along their ^1^H dimension in the presence of Ficoll 70 (Figure 1), indicating these proteins remain disordered under this crowded condition. ^1^H-^15^N HSQC spectra of ProTα and TC-1 in the presence of 400 g/L crowding agent had similar extents of peak dispersion as those collected in buffer or 160 g/L Ficoll conditions (Figures S1 and S2). Minor peak shifts between dilute and crowded conditions of some residues in TC-1 were observed (Figure 1B). To investigate the possibility that these spectral changes were due to the crowding agents binding to TC-1, we performed isothermal calorimetry (ITC) experiments, titrating 0.1 mM TC-1 into 160 g/L crowder solutions (Figure S3). These measurements were not indicative of specific interactions between TC-1 and Ficoll or Dextran 70 [72].

**Figure 2:**
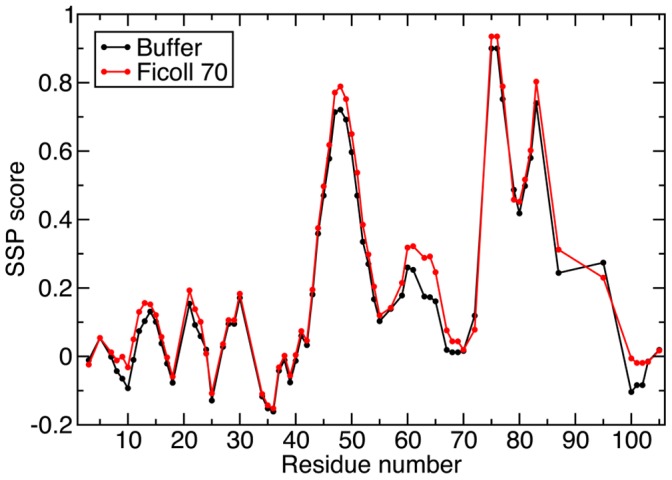
Secondary structure propensity (SSP) scores for TC-1 in the absence (black) and presence (red) of 160 g/L Ficoll 70. SSP scores were calculated on the basis of the assigned ^13^Cα and ^13^Cβ chemical shifts [46] using the SSP program [73]. The CBCA(CO)NH spectra was collected in 10 mM sodium acetate pH 5 in the absence and presence of 160 g/L Ficoll 70.

To determine if the chemical shift changes observed in the ^1^H-^15^N HSQC spectrum of TC-1 with 160 g/L of Ficoll 70 were the result of alteration of secondary structure, site-specific secondary structure propensities were determined based on the observed ^13^Cα and ^13^Cβ chemical shifts in the absence and presence of crowding agents using the SSP program [46], [73]. Residues in well-formed β-strand/extended or α-helical conformations are expected to yield SSP scores close to -1 and 1, respectively. Figure 2 shows the SSP score profiles of TC-1. While the N-terminal half of the protein is largely unstructured, three regions (D44-R53, K58-A64 and D73-T88) with high helical propensities (i.e. SSP scores >0.2) were found in the C-terminal part under both conditions. The results are consistent with our previous SSP analysis of TC-1 [46]. Based on the SSP scores reported here, it is apparent that the presence of crowding agents only leads to a minor increase in the helical propensity of the second helical region (K58-A64), while the other parts of the TC-1 structure are largely unaffected (Figure 2).

### Backbone ^15^N Spin Relaxation Measurements Under Crowded Conditions

**Figure 3:**
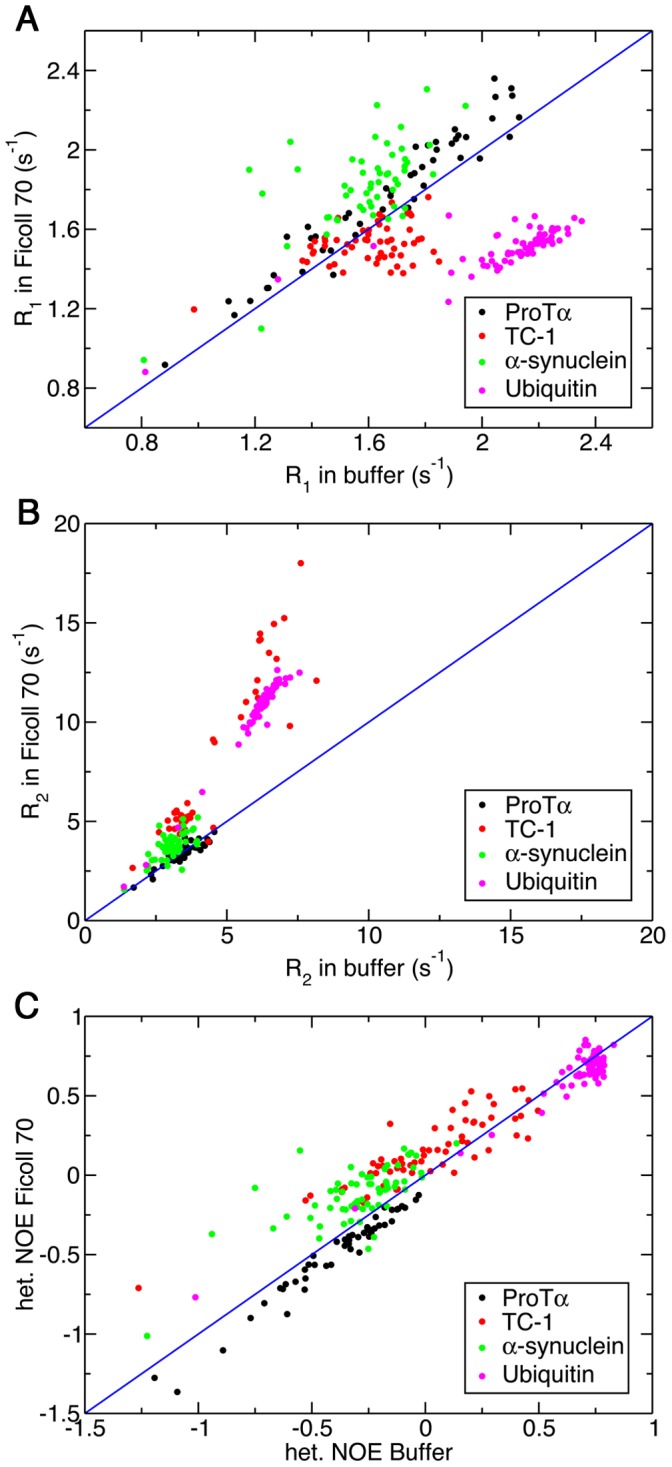
Backbone ^15^N relaxation measurements for ProTα, TC-1, α-synuclein and Ubiquitin in the absence and presence of 160 g/L Ficoll 70. Longitudinal relaxation rate, R_1_ (A), transverse relaxation rate, R_2_ (B) and steady-state ^1^H-^15^N NOE (C). ProTα (black), TC-1 (red), α-synuclein (green) and Ubiquitin (magenta) relaxation measurements were collected in 40 mM HEPES pH 6.8, 10 mM sodium acetate pH 5, 50 mM sodium phosphate pH 7 and 10 mM sodium acetate pH 5 respectively in the absence and presence of 160 g/L Ficoll 70. The blue line indicates a unitary slope.

The effects of macromolecular crowding on the dynamics of ProTα, TC-1, α-synuclein, and Ubiquitin were investigated with backbone ^15^N spin relaxation and ^1^H-^15^N NOE measurements. The results are shown in Figure 3. For the well-folded Ubiquitin, significant increases (decreases) in *R_2_* (*R_1_*) of residues are observed in the presence of 160 g/L of Ficoll 70. Because crowding does not alter the structure of Ubiquitin, judging from the ^1^H-^15^N HSQC spectra (Figure 1D), the changes in *R_2_* and *R_1_* are expected to be due to the increase in viscosity of the solution. Based on the *R_1_* and *R_2_* values, the overall rotational correlation time of Ubiquitin is estimated to increase from 4.3 to 8.0 ns upon addition of crowding agents [74]. Even though the molecular tumbling time was increased, crowding does not seem to have significant effects on the fast internal motion of this globular protein since the values of NOE were mostly unaffected by the addition of crowders.

**Figure 4:**
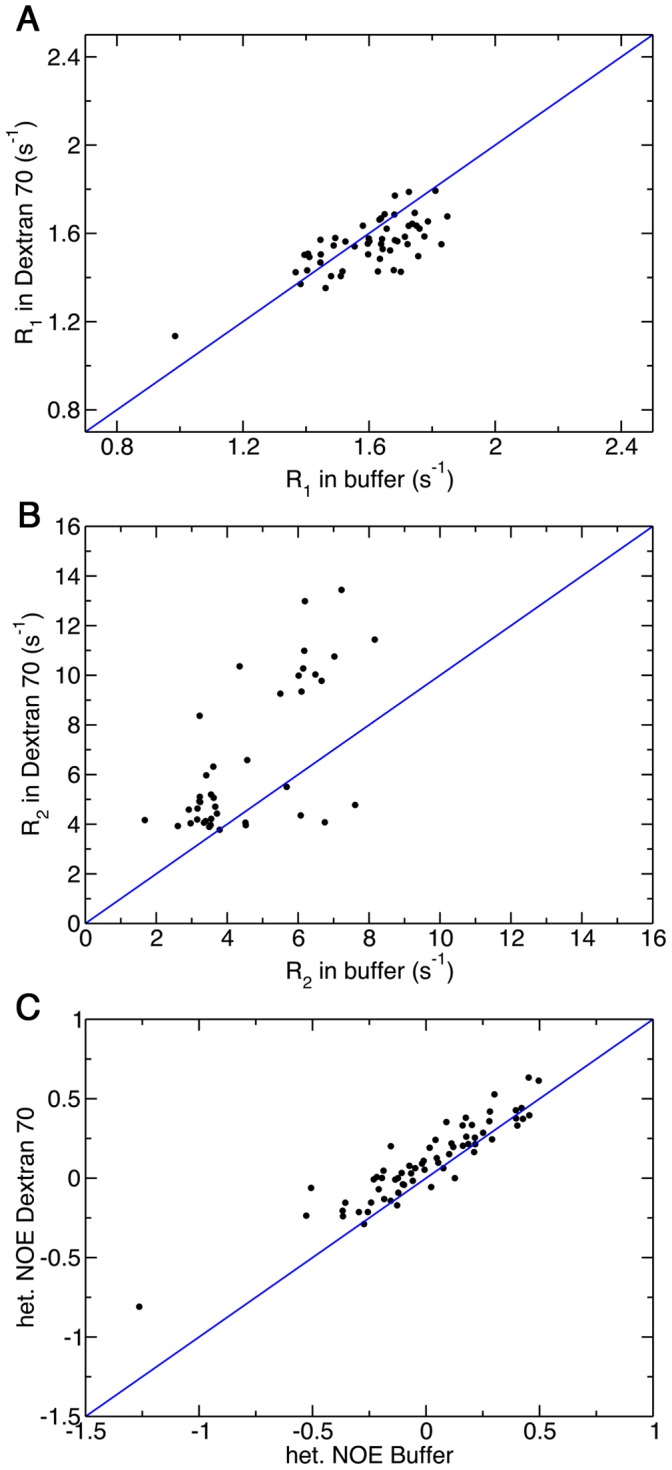
Backbone ^15^N relaxation measurements for TC-1 in the absence and presence of 160 g/L Dextran 70. Longitudinal relaxation rate, R_1_ (A), transverse relaxation rate, R_2_ (B) and steady-state ^1^H-^15^N NOE (C). The sample contained 10 mM sodium acetate pH 5 in the absence and presence of 160 g/L Dextran 70.

Unlike Ubiquitin, however, the increase in viscosity upon addition of 160 g/L of Ficoll 70 does not lead to dramatic changes in the observed *R_1_*, *R_2_* and NOE values of ProTα and α-synuclein (Figure 3). In particular, the value of *R_2_*, which is sensitive to the rotational correlation time, remains unchanged for most of the residues of ProTα upon addition of crowding agents. On the other hand, residues in different regions of TC-1 show differential responses to crowding. In particular, residues in the high helical propensity regions of TC-1 generally have decreased *R_1_* and increased *R_2_* relaxation rates in the presence of 160 g/L Ficoll 70 (Figure 3A and B), while *R_1_* and *R_2_* values of residues in the flexible N-terminal region show only minor changes. In addition, most of the residues in TC-1 also display slightly higher NOE values in the presence of 160 g/L of Ficoll 70 (Figure 3C). To ensure the observed changes in relaxation rates are not due to the particular crowding agent used, ^15^N relaxation experiments for TC-1 were also repeated with Dextran 70 as a crowder and the results were similar to that aforementioned (Figure 4). Figure S4 contains the *R_1_*, *R_2_* and NOE values for TC-1 in buffer and 160 g/L Ficoll and Dextran 70 plotted by residue number.

**Figure 5:**
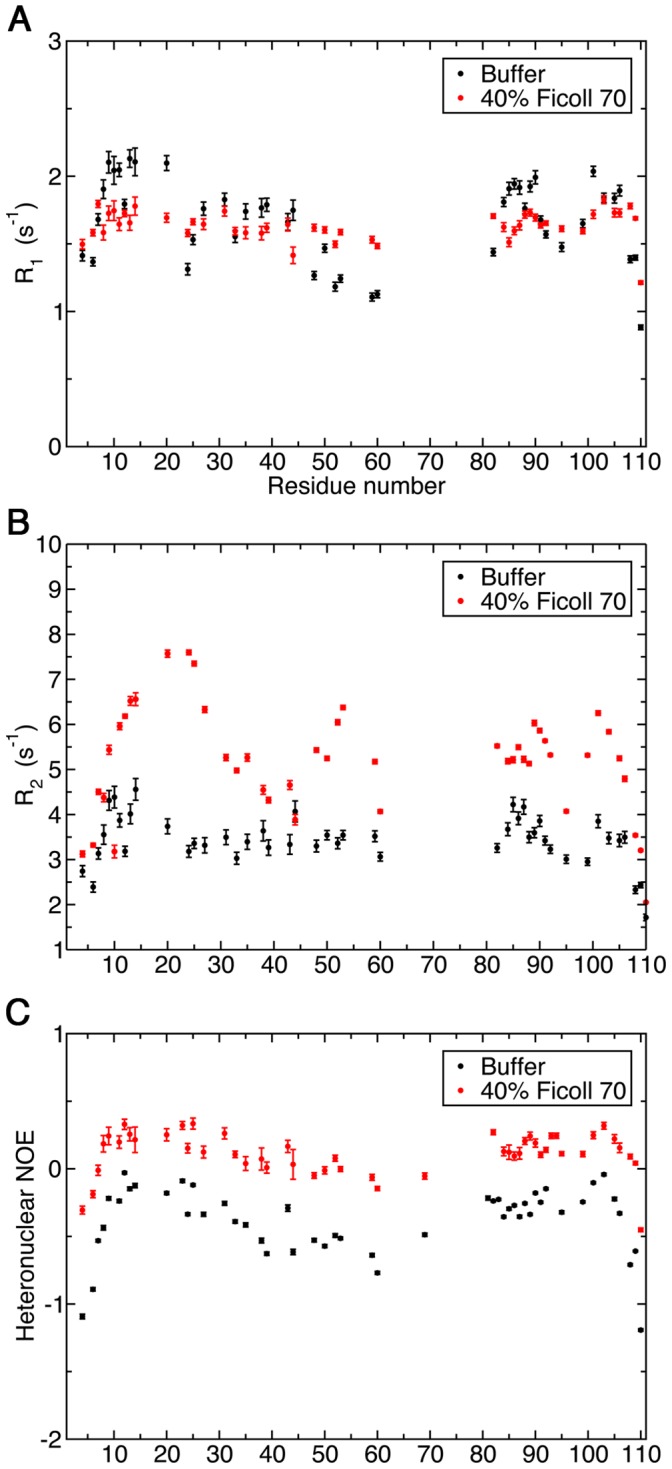
Backbone ^15^N relaxation measurements for ProTα in the absence and presence of 400 g/L Ficoll 70. Longitudinal relaxation rate, R_1_ (A), transverse relaxation rate, R_2_ (B) and steady-state ^1^H-^15^N NOE (C). The sample contained 0.3 mM ProTα in 50 mM NaPO_4_ pH 7, 100 mM NaCl and 1 mM DTT in the presence of 400 g/L Ficoll 70. For the sample without crowder, 40 mM HEPES pH 6.8 was used as the buffer.

Considerable changes in the relaxation rates were observed for ProTα when the extremely high concentration of crowding agent (400 g/L Ficoll 70) was used (Figure 5). In particular, most residues show higher *R_2_* values in the presence of 400 g/L Ficoll 70 compared to buffer conditions (Figure 5B). The largest changes are observed in the region around residues I12-R31. Interestingly, residues in that region also have less negative ^1^H-^15^N steady-state NOE values in buffer conditions, suggesting this segment is intrinsically more restricted in motion compared to the rest of the protein in the absence of crowders. Furthermore, NOE values were systematically higher for all residues under this crowded condition (Figure 5C).

### Model for Interpreting the Observed Relaxation Data

For well-folded globular proteins, the ^15^N *R_1_*, *R_2_*, and NOE measurements are commonly fitted to the Lipari-Szabo (LS) model-free model in order to extract the amplitude and correlation time of internal motion as well as the overall molecular tumbling time, which are denoted by the order parameter (*S^2^*), *τ_e_* and *τ_m_* in the spectral density function, respectively [75]. A modified LS model was later proposed by Clore and co-worker to fit the relaxation rates observed from flexible loop regions of a folded protein [76]. In this model, an extra term was introduced to the spectral density function of the original LS model to describe the internal motion occurring on a slower timescale. For disordered proteins, however, the timescale of large-amplitude local segmental motions can be close to the overall tumbling time, making the separation of these two contributions to the relaxation rates challenging [71], [77].

To establish a simple model to describe the dynamic behaviors of IDPs and correlate them to the observed relaxation parameters, autocorrelation functions of the backbone amide bond vectors were extracted from a 427-ns atomistic MD trajectory of ProTα. Autocorrelation functions of each residue (except the N-terminus and P34) were fitted to models with different numbers of exponential decay terms. Instead of using these models to back calculate the observed backbone ^15^N relaxation rates, which have been shown by many others to be a challenging task [78], [79], our aim is to establish a simple model to interpret the relaxation data we obtained.

**Figure 6:**
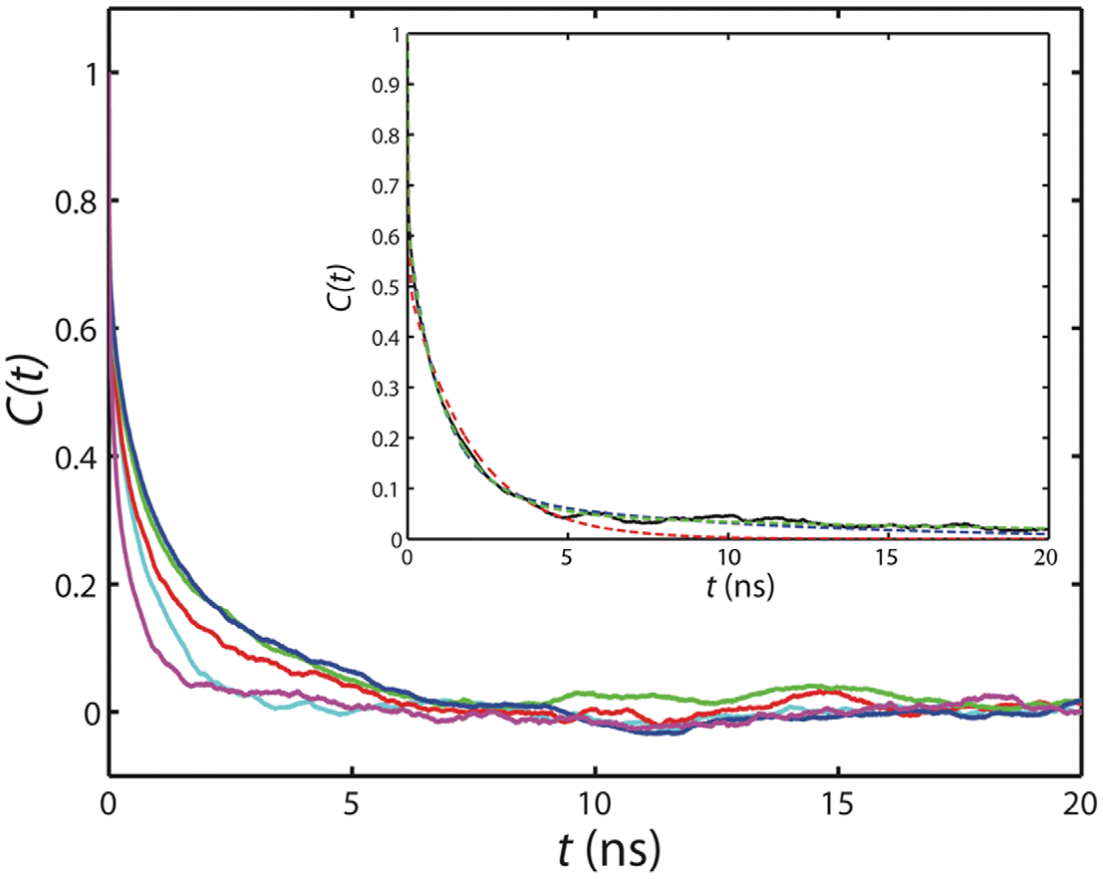
Correlation functions of selected backbone ^1^H-^15^N amide bond vectors (red: residue 2; green: residue 10; blue: residue 48; magenta: residue 57; cyan: residue 102) extracted from a 400 ns MD trajectory of ProTα. The inset shows the fitting of the autocorrelation function (solid black line) of residue 31 to 2- (red dash line), 3- (blue dash line), and 4-exponential decay curves (green dash line) as indicated in Equation 2. The blue and green dash lines overlay remarkably, and only start to deviate when t >15 ns.

Autocorrelation functions of individual amide bond vectors extracted from the MD simulation were fitted to the sum of two, three, or four exponential decay terms (Equation 2) in order to determine the best LS-like model that can be used to describe the backbone dynamics of highly disordered proteins such as ProTα. The autocorrelation functions of several residues are shown in Figure 6. In general, quick decreases in the autocorrelation functions are observed in the beginning, which are likely contributed from the librational motions (fast internal motions) [71], [75]. The fast decay is then followed by more gradual decreases in the autocorrelation functions, reflecting the existence of local motions on slower timescales (Figure 6). However, it is clear that residues in different positions of the protein display distinct autocorrelation profiles. Figure 6 (inset) shows typical fits of the autocorrelation functions to 2-, 3-, and 4-exponential decay terms. We found that for most of the residues, the equation with three exponential decay terms fits the autocorrelation function statistically better than that with only two terms. Increasing the number of exponential decay terms further (*i.e.* n = 4) does not result in dramatic decreases in the root mean square deviation of fitting (Figure S5). Additionally, for many residues, different *τ_i_* values obtained from the four-exponential fit are very close, indicating that the motion described by these terms cannot be discriminated. Because of these reasons, our analyses were focused on the three-exponential decay model (LS3 model; *n* = 3 in Equation 3), which is very similar to the modified LS-model described by Clore and coworkers [76].

**Figure 7:**
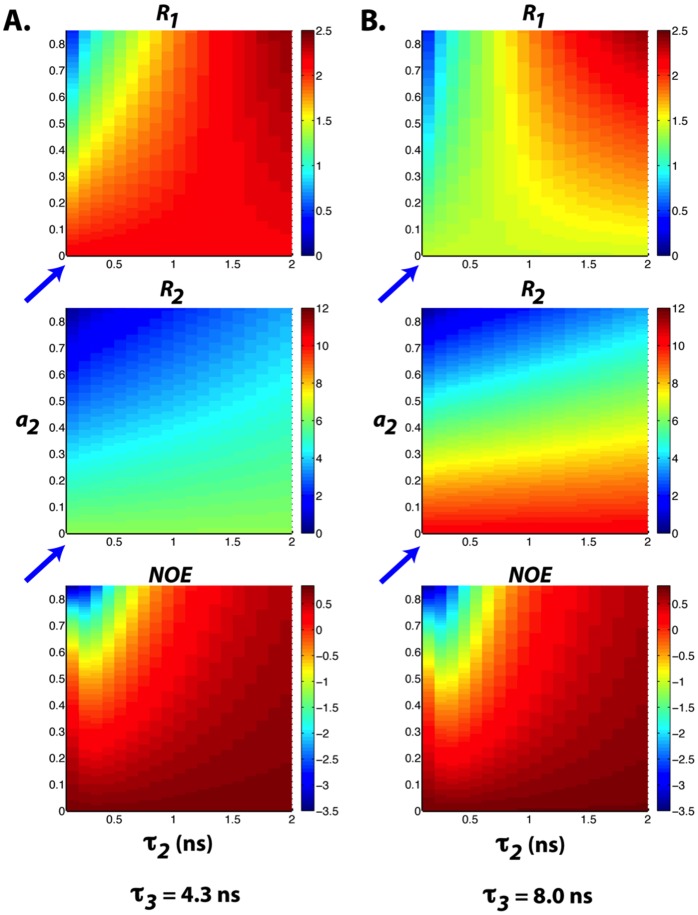
^15^N Relaxation parameters calculated using the LS-3 model with (a) *a_1_* = 0.15, *τ_1_* = 10 ps, *τ_3_* = 4.3 ns, *a_3_* = 1− *a_1_*–*a_2_* (b) *a_1_* = 0.15, *τ_1_* = 10 ps, *τ_3_* = 8.0 ns, *a_3_* = 1− *a_1–_a_2_. τ_2_* and *a_2_* values are indicated along the **x** **and y axes, respectively.** The slower internal motion is negligible when *a_2_* ∼ 0 (blue arrows).

The results of fitting the amide bond vector autocorrelation functions to three-exponential decay terms are summarized in Table 1. To illustrate how the fluctuations in amplitude and timescale of motions translate to the observed relaxation rate changes, ^15^N *R_1_*, *R_2_*, and ^1^H-^15^N steady-state NOE values were calculated using the LS3 model with different values of *a_i_* and *τ_i_*. We first apply this model to Ubiquitin. To simulate the relaxation rates of Ubiquitin, we assumed that the fast internal motion of this rigid protein is not altered upon crowding. By fixing the amplitude and correlation time of fast internal motion (*a_1_* and *τ_1_*) to 0.15 and 10 ps, respectively, the significant increase (decrease) in the measured *R_2_* (*R_1_*) relaxation rates of Ubiquitin in the presence of 160 g/L of Ficoll 70 can be reproduced by changing *τ_3_* (the overall tumbling time) from 4.3 to 8 ns, assuming that the slower segmental motion can be neglected (i.e. *a_2_* ∼ 0; blue arrows) (Figure 7).

**Table 1:** Averaged values and the standard deviations of fitted parameters of LS-3 model.

	*i* = 1	*i* = 2	*i* = 3
*τ_i_* (ps)	7±9	419±454	3400±5700
*a_i_*	0.37±0.09	0.36±0.12	0.27±0.17

average±standard.

**Figure 8:**
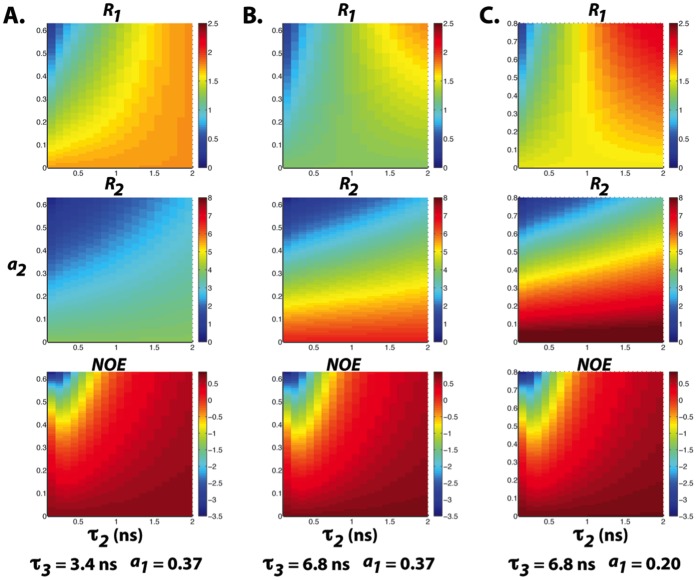
^15^N Relaxation parameters calculated using the LS-3 model with (a) *a_1_* = 0.37, *τ_1_* = 7 ps, *τ_3_* = 3.4 ns, *a_3_* = 1− *a_1–_a_2_* (b) *a_1_* = 0.37, *τ_1_* = 7 ps, *τ_3_* = 6.8 ns, *a_3_* = 1− *a_1–_a_2_* and (c) *a_1_* = 0.20, *τ_1_* = 7 ps, *τ_3_* = 6.8 ns, *a_3_* = 1− *a_1–_a_2_,* respectively. *τ_2_* and *a_2_* values are indicated along the x and y axes, respectively.

We have also simulated the dependence of the ^15^N *R_1_*, *R_2_*, and steady-state NOE values of ProTα on the values of *a_i_* and *τ_i_*. Since ProTα remains disordered under crowded conditions and the observed NOEs are significantly smaller than what are expected for a folded protein of similar molecular weight (Figure 5), it is reasonable to assume that large amplitude of fast internal motion persists. Figure 8A illustrates that with *a_1_* = 0.37, *τ_1_* = 7 ps, *τ_2_* ∼500 ps, and *τ_3_* = 3.4 ns, a wide distribution of NOE values can be expected with the variation of the amplitude of segmental motion (value of *a_2_*). Meanwhile, *R_2_* is predicted to be not very sensitive to the fluctuation in *a_2_* (*R_2_* ∼ 2–4 s^-1^). These observations agree qualitatively with the distributions of experimental relaxation rates measured under buffer conditions (Figure 5).

On the other hand, almost all residues of ProTα have the *R_2_* and NOE increased at the high concentration of crowding agents (∼400 g/L of Ficoll 70), while the variation of *R_1_* along the protein sequence diminished. Based on the LS3 model, these trends can be explained by the increase in the correlation times of the slow local segmental motions. With *τ_2_* increases from 500 to 1000 ps and the value of *τ_3_* doubled (Figure 8B), *R_2_* values can increase to ∼6 s^−1^ and many NOEs will turn positive. The simulated relaxation rates further match the experimentally observed values, especially for the *R_1_* values, if we assume that the amplitude of fast internal motion is reduced in a highly crowded environment (i.e. *a_1_* = 0.2; Figure 8C).

**Figure 9:**
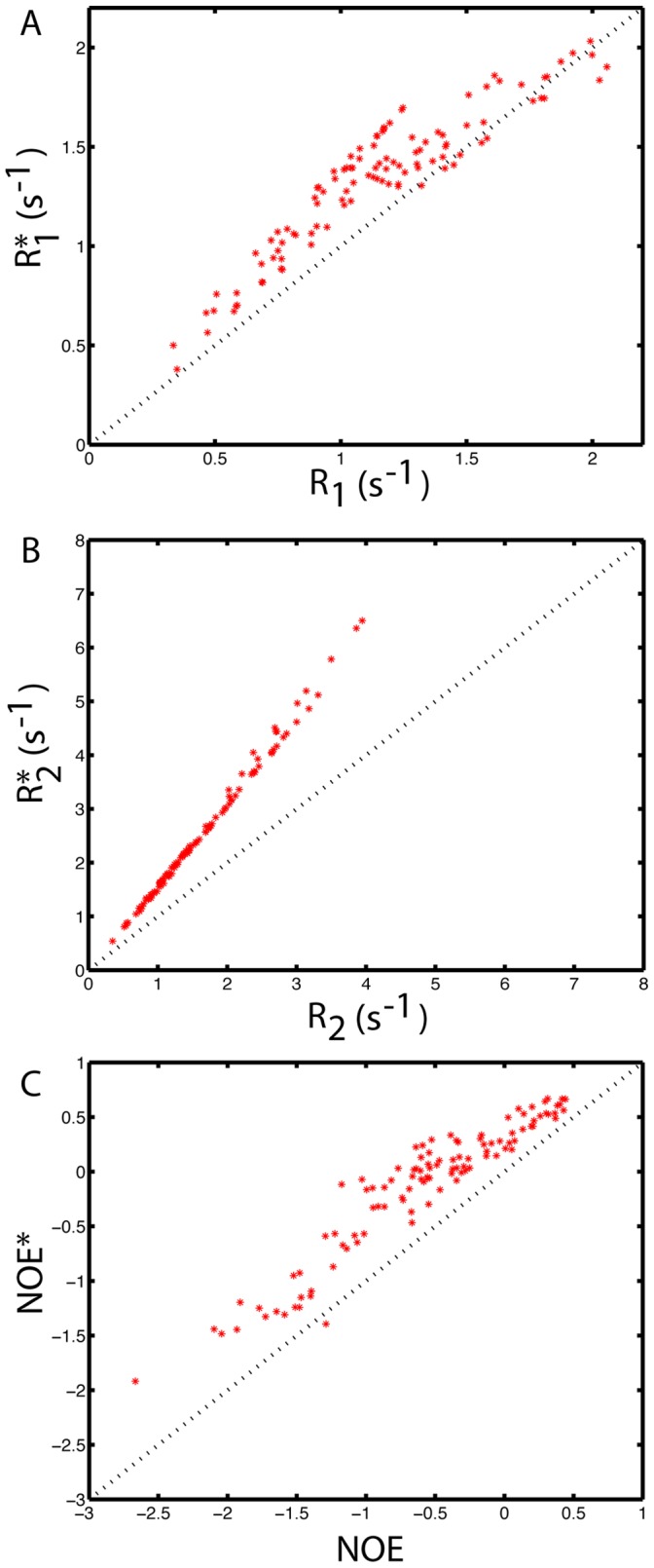
Plots of the simulated relaxation data of ProTα before and after correlation time adjustments. ^15^N *R_1_*, *R_2_*, and steady-state *NOE* values of ProTα were simulated based on the amplitudes and correlation times of motions extracted from the MD simulation using the LS3 model. R_1_*, R_2_*, and NOE* are the relaxation data predicted by scaling the correlation times of the slow motions (τ_2_ and τ_3_) by the same factor as the Ubiquitin tumbling time changes to account for the increase in viscosity.

Finally, based on the amplitudes and correlation times of motions on different timescales (fitted *a_i_* and *τ_i_* values of autocorrelation functions) extracted from the MD simulation, we have simulated the ^15^N *R_1_*, *R_2_*, and steady-state NOE values of ProTα. The relaxation parameters in the presence of 160 g/L of Ficoll 70 were then predicted by scaling the correlation time of the slow motions (*τ_2_* and *τ_3_*) by the same factor (i.e. 1.86) as the Ubiquitin tumbling time changes to account for the increase in viscosity. Figure 9 shows the plots of the simulated relaxation data before and after the correlation time adjustments. The result indicates that in the presence of 160 g/L of Ficoll 70, the *R_1_*, *R_2_*, and NOE of ProTα were expected to systematically increase if the correlation times of the slow motions were increased by viscosity. However, these changes were observed experimentally only in the presence of 400 g/L of Ficoll 70. Again, the simulated data suggest that the timescale of local segmental motions were slowed down only at a very high concentration of crowders.

## Discussion

We have investigated the effects of macromolecular crowding on the dynamics of three IDPs, ProTα, TC-1 and α-synuclein, with different extents of residual structure using NMR spectroscopy. This complements several recent studies of macromolecular crowding effects on the structure and dynamics of IDPs [34], [35], [80]. We used Ficoll 70 and Dextran 70 as crowding agents, which are commonly used to mimic excluded volume effects [7], [17], [18], [72]. These polymers are inert and do not interact nonspecifically with proteins. In contrast, the use of polyethylene glycol as a crowding agent is discouraged, due to attractive interactions with proteins [7], [72].

The IDPs examined here all had narrow dispersion of peaks along the ^1^H dimension in the ^1^H-^15^N HSQC spectra compared to the well-folded Ubiquitin, both in the absence and presence of crowding agents, suggesting that they remain disordered in the crowded environments. Interestingly, for the partially disordered TC-1, a minor increase of the helical propensity was observed only in the relatively structured region in the presence of Ficoll 70. This indicates that the crowded environment may have differential effects on the partially structured regions and the highly disordered parts of the protein. Increased helical content in the presence of crowding agent has also been observed for the Flavodoxin [10]. Stagg et al. reported that the far-UV CD signal of Flavodoxin at the helical signature wavelength (222 nm) increases by about 10% in the presence of 200 g/L of Ficoll 70; however, a less dramatic effect of crowding in the denatured state was observed.

Site-specific changes in the protein flexibility of ProTα and TC-1 have been characterized by using ^15^N NMR spin relaxation experiments. In particular, we focused on the highly disordered ProTα since this protein produces NMR data with reasonable signal to noise ratio even at high concentration of Ficoll 70 (400 g/L). It is noteworthy that besides the excluded volume effect, the presence of high concentrations of crowding agents also inevitably increases the viscosity of the solution [12], [34]. This adds a layer of complexity to the interpretation of spin relaxation data. The viscosity effect is reflected in the systematic increase in the ^15^N *R_2_* rates of Ubiquitin in the presence of 160 g/L Ficoll 70, while the values of NOE were mostly unaffected. Similar results were obtained by Simorellis & Flynn [81]. They showed that encapsulation of Ubiquitin in a confined environment only has very minor effects on the protein backbone dynamics.

Intriguingly, the increase in viscosity did not cause significant changes in the ^15^N *R_2_* of intrinsically disordered ProTα under the same conditions. To have a better understanding of our relaxation data, we performed an MD simulation (∼400 ns) on ProTα to investigate its dynamic behaviors. Although MD simulations in the presence of atomistically represented crowders are not currently practical (because of the large number of atoms these molecules contain and the long time scales such molecules need for diffusion), our simulation facilitated the development of a simple model to correlate the observed changes in relaxation rates to the alteration in protein motions under crowding conditions. While the LS3 model proposed here might not be sufficient to represent the complicated dynamics of IDPs, it provides insights into interpreting the relaxation measurements.

Based on the experimental and simulation results, we conclude that even though crowded environments can slow down the timescale of local segmental motions in the highly disordered ProTα, it still retains a certain level of flexibility at high concentrations of Ficoll 70. Based on the observed *R_2_* rates (Figure 5B), however, it is apparent that a few regions of ProTα become more structured at high concentration of crowders. Interestingly, some of these regions overlap or are close to known target-binding motifs of ProTα. For instance, residues 39–54 are involved in mediating the interaction with the Kelch domain of Keap1 in the oxidative stress response [82] while the caspase-3 cleavage site of ProTα is located around residue 100 [83]. Because the dynamics of IDPs can have significant impacts on their target recognitions [60], this observation has a strong biological implication of how this class of proteins functions in crowded cellular environments.

We are aware that while Ficoll and Dextran may be suitable agents to mimic the crowded cellular environment, combining different crowding agents with varying physical characteristics (sizes, shapes, charges, etc) may more accurately represent the *in vivo* environment [2], [7], [84], [85]. Therefore, extending the current studies by using other crowding agents with different sizes and chemical properties are required to further our understanding of the macromolecular crowding effects on IDPs. These in vitro studies together with the recently developed in cell NMR techniques [86]–[92] will hopefully provide further insights into understanding the environmental effects on IDP structure and functions.

## Supporting Information

Figure S1 **^1^H-^15^N HSQC spectrum of ProTα in 400 g/L Ficoll 70.** The sample contained 0.3 mM ProTα in 50 mM NaPO_4_ pH 7, 100 mM NaCl and 1 mM DTT.
(PDF)

Figure S2 **^1^H-^15^N HSQC spectra of TC-1 in 400 g/L Ficoll 70 and Dextran 70.** The samples contained 0.2 mM TC-1 in 10 mM sodium acetate pH 5 and 400 g/L Ficoll 70 (A) or Dextran 70 (B).
(PDF)

Figure S3 **ITC profiles of TC-1 titrations into crowded solutions.** Buffer (10 mM sodium acetate pH 5) alone or containing 0.1 mM TC-1 was titrated into the cell, containing 160 g/L Ficoll (A) or Dextran 70 (B) in the same buffer. 10 µL injections were used with 120-second delays.
(PDF)

Figure S4 ***R_1_*** **,** ***R_2_*** **and NOE values for TC-1 in buffer and 160 g/L Ficoll 70 and Dextran 70 plotted by residue number.** The samples contained 10 mM sodium acetate pH 5 in absence and presence of 160 g/L Ficoll 70 or Dextran 70.
(PDF)

Figure S5 **Comparison of the fitting of autocorrelations to 2-, 3-, and 4-exponential decay curves.** Blue: *F*-ratios calculated from the χ^2^ and degrees of freedom of 2- and 3-exponential models; Red: *F*-ratios calculated from the χ^2^ and degrees of freedom of 3- and 4-exponential models (Equation 4).
(PDF)
