# Supplementary material for: Effects of Molecular Crowding on the Dynamics of Intrinsically Disordered Proteins
Source: PLoS One. 2012 Nov 26;7(11):e49876. doi: 10.1371/journal.pone.0049876 (PMC3506533; doi:10.1371/journal.pone.0049876)
Supplement: Figure S1 — 1H-15N HSQC spectrum of ProTα in 400 g/L Ficoll 70. The sample contained 0.3 mM ProTα in 50 mM NaPO4 pH 7, 100 mM NaCl and 1 mM DTT. (PDF) [file pone.0049876.s001.pdf]

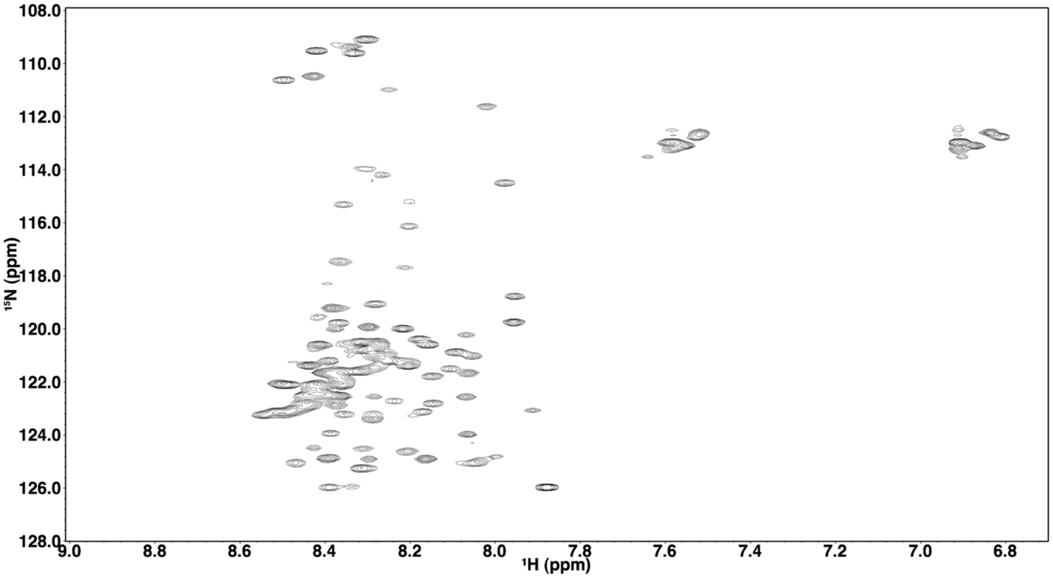

Figure S1. ^{1}H-^{15}N HSQC spectrum of ProTα in 400 g/L Ficoll 70. The sample contained 0.3 mM ProTα in 50 mM $NaPO_4$ pH 7, 100 mM NaCl and 1 mM DTT.
