# Supplementary material for: Effects of Molecular Crowding on the Dynamics of Intrinsically Disordered Proteins
Source: PLoS One. 2012 Nov 26;7(11):e49876. doi: 10.1371/journal.pone.0049876 (PMC3506533; doi:10.1371/journal.pone.0049876)
Supplement: Figure S2 — 1H-15N HSQC spectra of TC-1 in 400 g/L Ficoll 70 and Dextran 70. The samples contained 0.2 mM TC-1 in 10 mM sodium acetate pH 5 and 400 g/L Ficoll 70 (A) or Dextran 70 (B). (PDF) [file pone.0049876.s002.pdf]

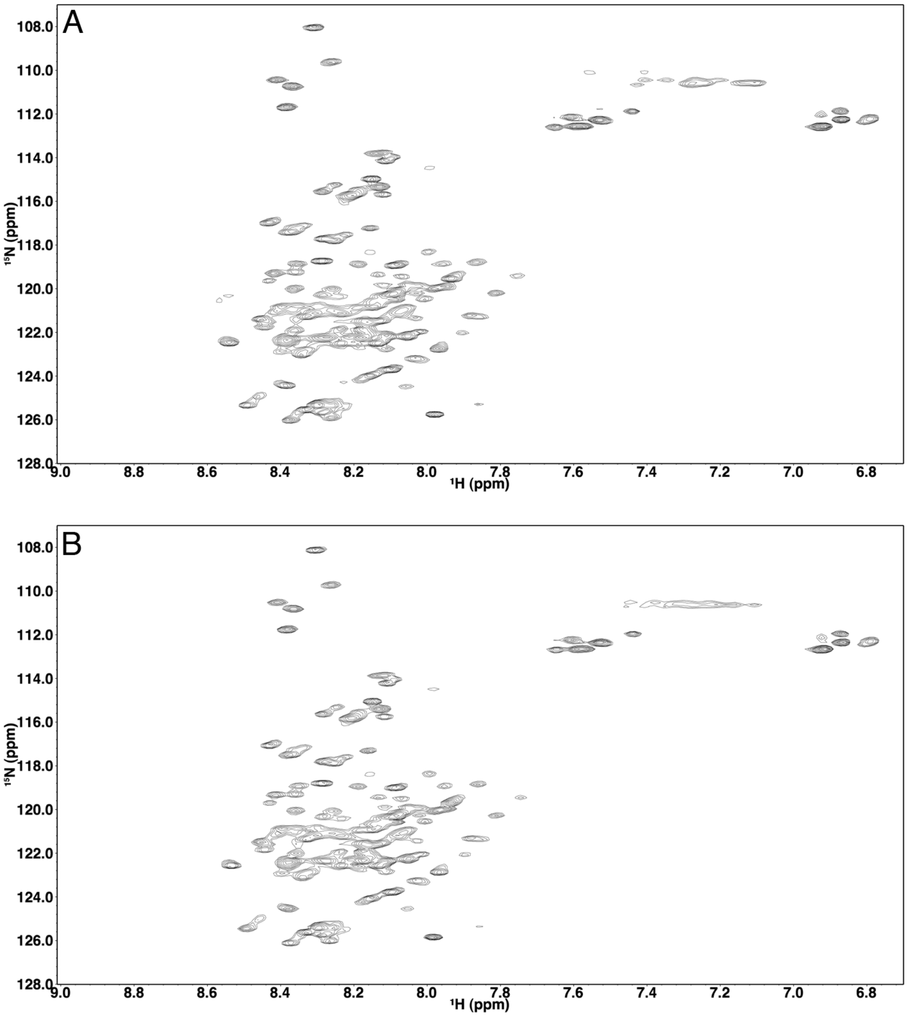

Figure S2. ^{1}H-^{15}N HSQC spectra of TC-1 in 400 g/L Ficoll 70 and Dextran 70. The samples contained 0.2 mM TC-1 in 10 mM sodium acetate pH 5 and 400 g/L Ficoll 70 (A) or Dextran 70 (B).
