# Supplementary material for: Effects of Molecular Crowding on the Dynamics of Intrinsically Disordered Proteins
Source: PLoS One. 2012 Nov 26;7(11):e49876. doi: 10.1371/journal.pone.0049876 (PMC3506533; doi:10.1371/journal.pone.0049876)
Supplement: Figure S3 — ITC profiles of TC-1 titrations into crowded solutions. Buffer (10 mM sodium acetate pH 5) alone or containing 0.1 mM TC-1 was titrated into the cell, containing 160 g/L Ficoll (A) or Dextran 70 (B) in the same buffer. 10 µL injections were used with 120-second delays. (PDF) [file pone.0049876.s003.pdf]

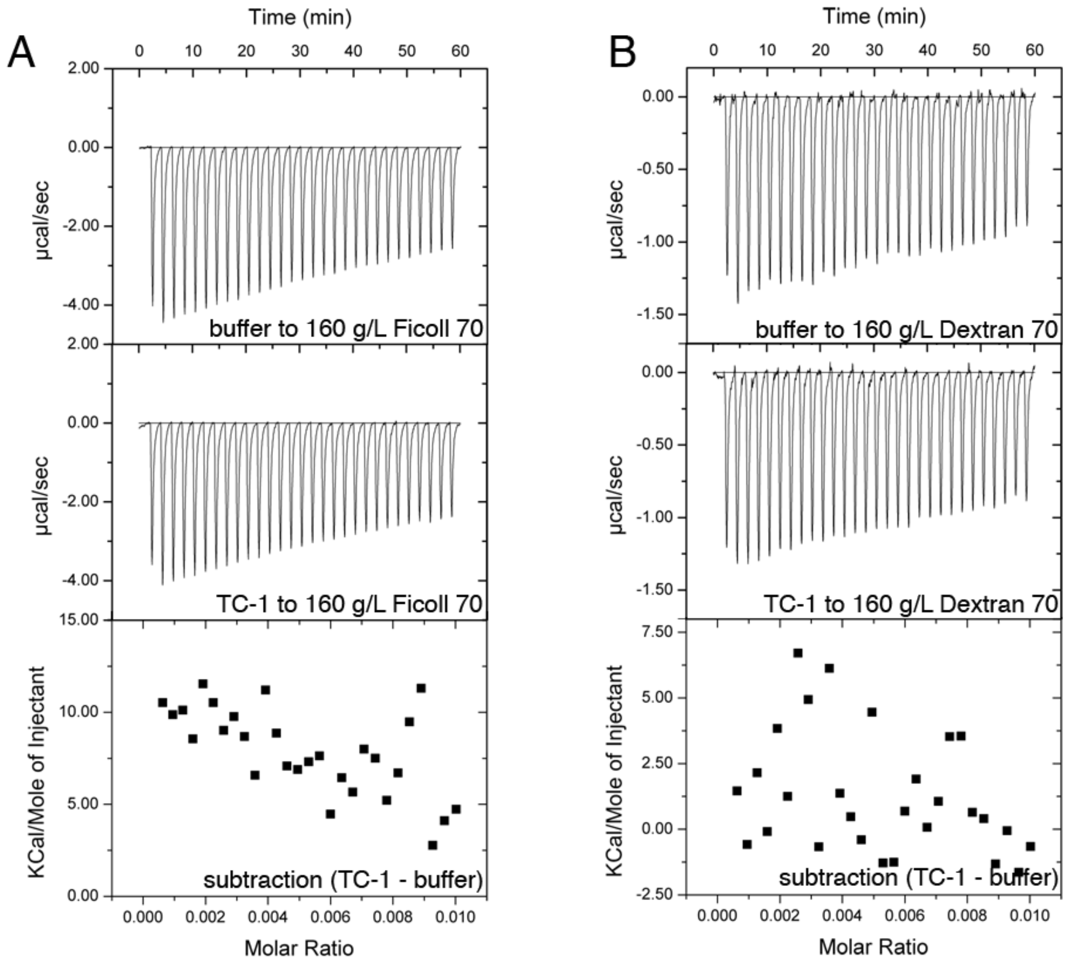

Figure S3. ITC profiles of TC-1 titrations into crowded solutions. Buffer (10 mM sodium acetate pH 5) alone or containing 0.1 mM TC-1 was titrated into the cell, containing 160 g/L Ficoll (A) or Dextran 70 (B) in the same buffer. 10 μL injections were used with 120-second delays.
