# Supplementary material for: Effects of Molecular Crowding on the Dynamics of Intrinsically Disordered Proteins
Source: PLoS One. 2012 Nov 26;7(11):e49876. doi: 10.1371/journal.pone.0049876 (PMC3506533; doi:10.1371/journal.pone.0049876)
Supplement: Figure S4 — R1 , R2 and NOE values for TC-1 in buffer and 160 g/L Ficoll 70 and Dextran 70 plotted by residue number. The samples contained 10 mM sodium acetate pH 5 in absence and presence of 160 g/L Ficoll 70 or Dextran 70. (PDF) [file pone.0049876.s004.pdf]

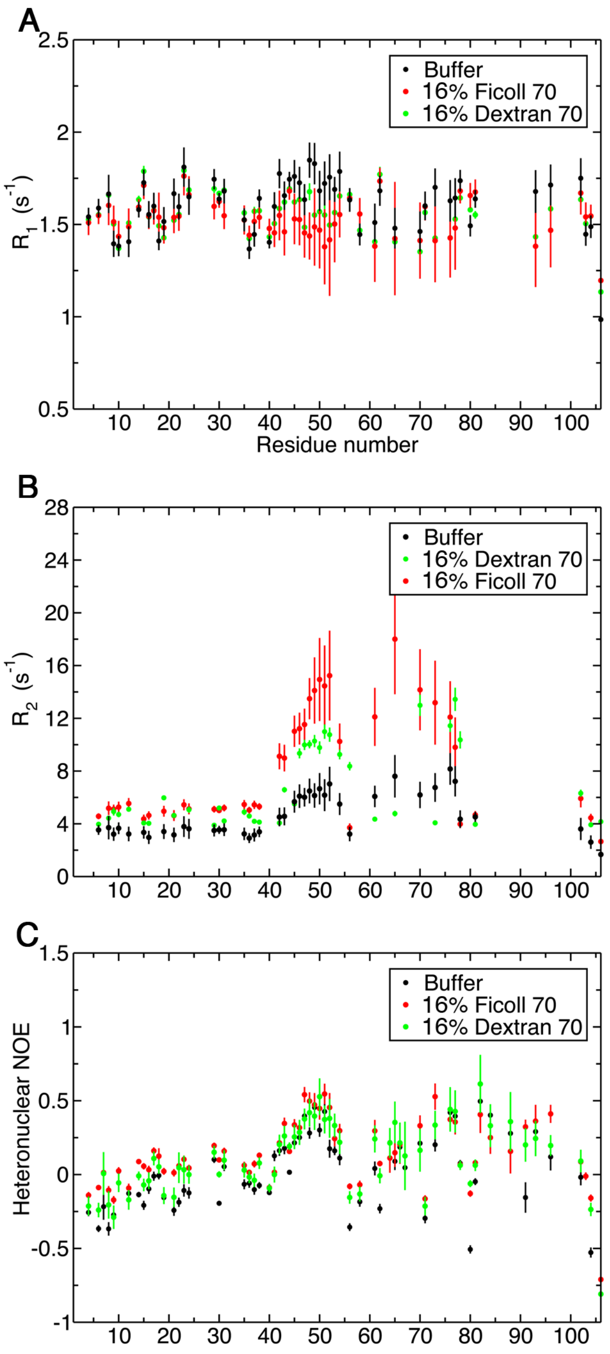

Figure S4. R_1, R_2 and NOE values for TC-1 in buffer and 160 g/L Ficoll 70 and Dextran 70 plotted by residue number. The samples contained 10 mM sodium acetate pH 5 in absence and presence of 160 g/L Ficoll 70 or Dextran 70.
