# Supplementary material for: Effects of Molecular Crowding on the Dynamics of Intrinsically Disordered Proteins
Source: PLoS One. 2012 Nov 26;7(11):e49876. doi: 10.1371/journal.pone.0049876 (PMC3506533; doi:10.1371/journal.pone.0049876)
Supplement: Figure S5 — Comparison of the fitting of autocorrelations to 2-, 3-, and 4-exponential decay curves. Blue: F-ratios calculated from the χ2 and degrees of freedom of 2- and 3-exponential models; Red: F-ratios calculated from the χ2 and degrees of freedom of 3- and 4-exponential models (Equation 4). (PDF) [file pone.0049876.s005.pdf]

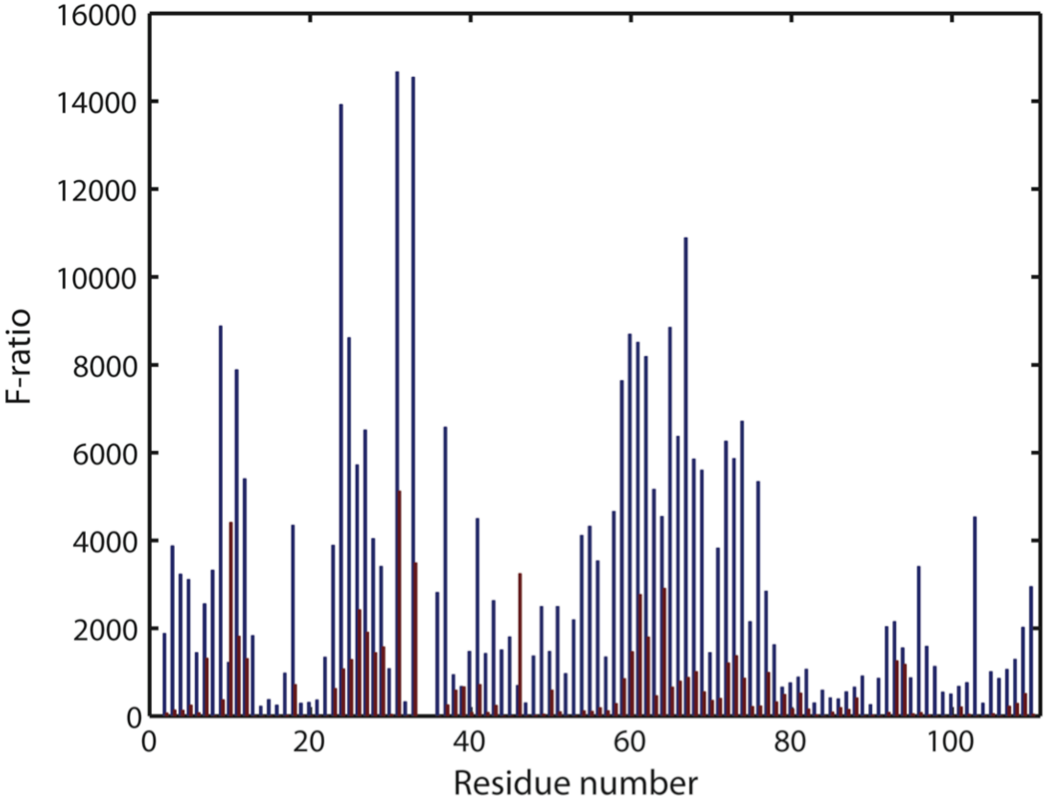


Figure S5. Comparison of the fitting of autocorrelations to 2-, 3-, and 4-exponential decay curves. Blue: *F*-ratios calculated from the $\chi^2$ and degrees of freedom of 2- and 3-exponential models; Red: *F*-ratios calculated from the $\chi^2$ and degrees of freedom of 3- and 4-exponential models (Equation 4).
